# Inter-Individual Differences in the Oral Bacteriome Are Greater than Intra-Day Fluctuations in Individuals

**DOI:** 10.1371/journal.pone.0131607

**Published:** 2015-06-29

**Authors:** Yukuto Sato, Junya Yamagishi, Riu Yamashita, Natsuko Shinozaki, Bin Ye, Takuji Yamada, Masayuki Yamamoto, Masao Nagasaki, Akito Tsuboi

**Affiliations:** 1 Department of Integrative Genomics, Tohoku Medical Megabank Organization, Tohoku University, 2–1, Seiryo-machi, Aoba-ku, Sendai, 980–8573, Japan; 2 Division of Collaboration and Education, Research Center for Zoonosis Control, Hokkaido University, North 20, West 10 Kita-ku, Sapporo, Hokkaido, 001–0020, Japan; 3 Global Station for Zoonosis Control, GI-CoRE, Hokkaido University, North 20, West 10 Kita-ku, Sapporo, Hokkaido, 001–0020, Japan; 4 Department of Bioinformation, School and Graduate School of Bioscience and Biotechnology, Tokyo Institute of Technology, 4259 Nagatsutacho, Midoriku, Yokohama, Kanagawa, 226–8501, Japan; 5 Department of Medical Biochemistry, Tohoku Medical Megabank Organization, Tohoku University, 2–1, Seiryo-machi, Aoba-ku, Sendai, 980–8573, Japan; 6 Department of Community Medical Supports, Tohoku Medical Megabank Organization, Tohoku University, 2–1, Seiryo-machi, Aoba-ku, Sendai, 980–8573, Japan; Estacion Experimental del Zaidin - CSIC, SPAIN

## Abstract

Given the advent of massively parallel DNA sequencing, human microbiome is analyzed comprehensively by metagenomic approaches. However, the inter- and intra-individual variability and stability of the human microbiome remain poorly characterized, particularly at the intra-day level. This issue is of crucial importance for studies examining the effects of microbiome on human health. Here, we focused on bacteriome of oral plaques, for which repeated, time-controlled sampling is feasible. Eighty-one supragingival plaque subjects were collected from healthy individuals, examining multiple sites within the mouth at three time points (forenoon, evening, and night) over the course of 3 days. Bacterial composition was estimated by 16S rRNA sequencing and species-level profiling, resulting in identification of a total of 162 known bacterial species. We found that species compositions and their relative abundances were similar within individuals, and not between sampling time or tooth type. This suggests that species-level oral bacterial composition differs significantly between individuals, although the number of subjects is limited and the intra-individual variation also occurs. The majority of detected bacterial species (98.2%; 159/162), however, did not fluctuate over the course of the day, implying a largely stable oral microbiome on an intra-day time scale. In fact, the stability of this data set enabled us to estimate potential interactions between rare bacteria, with 40 co-occurrences supported by the existing literature. In summary, the present study provides a valuable basis for studies of the human microbiome, with significant implications in terms of biological and clinical outcomes.

## Introduction

The microbiome is one of the important factors in human health, playing a role in numerous diseases including cardiovascular disease, inflammatory rheumatism, type II diabetes, colorectal carcinoma, psoriasis, colitis, and inflammatory bowel disease [1–5]. The human microbiome is now comprehensively analyzed by massively parallel DNA sequencing with complementary use of more traditional methods such as cultivation- and microscopy-based approaches [6–10]. PCR amplification and the sequencing of 16S rRNA fragments present in human samples (*e*.*g*., oral plaque, saliva, and feces) enable comparative analyses of bacterial compositions between patients and controls. Such a situation allows for a comprehensive assessment of microbial species associated with various human diseases, including rare and unculturable species.

While the microbiome composition of most of human body sites has been examined, little is known in regard to the stability and fluctuations of the human microbiome over short periods of time. Several studies have addressed temporal variations in the composition of oral, gut, and skin bacteria [11–14] and oral fungi [14] over the course of several days to year (365 days), revealing a relatively stable microbial environment at these time scales. On the other hand, significant intra-day fluctuations are observed in the abundance and activity of numerous host biomolecules with circadian oscillations affecting several hundred mRNAs in mice [15] and in about 150 of 2,000 metabolites in humans [16]. It is therefore particularly important to determine whether the human microbiome exhibits such periodic fluctuations over the course of a day, similarly to other omics dynamics, as recently reported for the gut microbiome [17]–[18].

In this study, we address the intra-day stability and possible fluctuations in the human microbiome by focusing on oral bacteria, for which a highly specific, time-controlled sample collection is possible. A total of 81 supragingival plaques were collected from three healthy individuals, and the bacterial composition was estimated using amplicon sequencing of the partial bacterial 16S rRNA V4 region. Samples were taken from three tooth types (molar, premolar, and incisor) across nine time points (forenoon, evening, and night) for 3 days. From these data, we conducted species-level taxonomic assignments to perform a comparative analysis of bacterial compositions over time, including an exploration of possible co-occurring and co-fluctuating combinations of bacterial species by pairwise correlation analysis. On the basis of these periodic sampling and systematic analysis, the present study provides the first insight into the intra-day stability of the human oral microbiome, with implications for the designing of future microbiome and human health studies.

## Results

### Oral bacterial 16S rRNA sequencing and taxonomic assignments

Using the Illumina MiSeq platform, we obtained a total of 3,886,475 paired-end sequences of partial 16S rRNA V4 region from 81 oral plaque samples. Samples were comprised of supragingival plaques from three types of lower teeth (incisor, premolar, and molar) of three individuals collected at three consecutive time points (forenoon, evening, and night) on three non-consecutive days. An average of 47,981 reads were obtained per sample (± 1,002 S.E., ranging from 25,697 to 85,040 reads; S1 Table; DDBJ Sequence Read Archive (DRA) accession number: DRA003069). The overall quality of sequence data was confirmed, with average Phred scores of 36.2 and 35.6 for forward and reverse reads, respectively. No technical errors were found in the DNA sequencing and nucleotide-base calling procedures, as determined using a quality-check analysis by FastQC (http://www.bioinformatics.babraham.ac.uk/projects/fastqc/) and Sugar [19]. After quality filtering, paired-end read assembly, and primer trimming, an average of 85.4% of raw sequences remained (mean 41,228; range of 2,062–62,153), with the majority of sequences 258–259 base pairs (bp) in length. Most samples exhibited > 15,000 high-quality reads, though two incisor contained fewer usable sequences (< 5,000; see S1 Table).

Similarity-based analysis of sequencing reads revealed a total of 162 known bacterial species across all 81 samples, with an average of 125 bacterial species per person (mean 124.7 ± 8.65 S.E.; 108, 129, and 137 total species for individuals 1, 2, and 3, respectively). These numbers were comparable to those reported previously [20], [21]. This analysis included the clustering of sequences into groups with ≥ 99% sequence-similarity to achieve species-level resolution, followed by BLASTn analysis against the Human Oral Microbiome Database (HOMD) [22], [23]. Known bacterial species accounted for an average of 87.6% of sequences per subject (ranging from 62.7% to 95.8%) based on the top BLASTn hit for each read. Four technical replicates were performed for both DNA sequencing and taxonomic assignments, with Pearson’s correlation coefficients ranging from 0.962 to 0.998 (*p* < 0.001) between estimated bacterial compositions (S1 Fig).

### Bacterial diversity in oral plaques among intra-day time points

**Fig 1:**
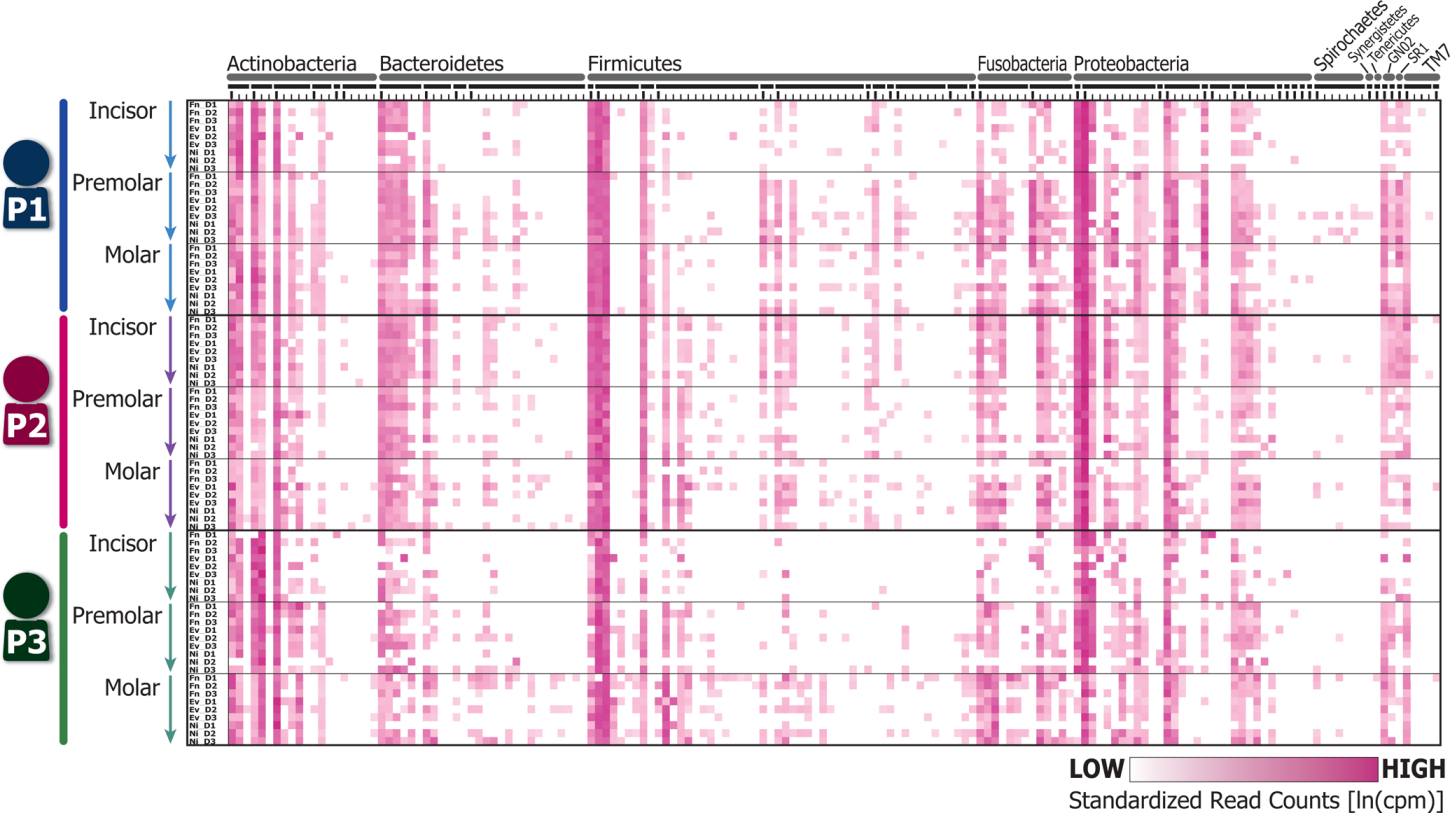
Schematic representation of relative sequence read counts of oral bacterial species detected. Columns indicate the 162 bacterial species identified in this study. The upper horizontal gray and black bars indicate phyla and families, respectively. Rows indicate each plaque samples; P1, P2, and P3 indicate subjects 1–3, respectively. Fn, forenoon; Ev, evening; Ni, night; D1, D2, and D3 indicate day1–3, respectively. Matrices colored in magenta shading show relative sequence read counts standardized to logarithm of counts per million (cpm) reads across all samples. Legend of the horizontal axis is provided as S5 Fig.

The estimated species-level bacterial compositions were largely consistent for major bacterial species across subjects, time points, and location, while relatively less common species varied between subjects (Fig 1). Among the major species, several were detected at larger numbers (> 5,000 counts per million [cpm] reads on average across all samples), including *Corynebacterium matruchotii*, *Corynebacterium durum*, *Rothia aeria*, and *Actinomyces sp*. (1st, 2nd, 4th, and 7th columns of the phylum Actinobacteria in Fig 1), *Bergeyella sp*. (1st column of the phylum Bacteroidetes in Fig 1), *Streptococcus sanguinis* and *Streptococcus sp*. (2nd and 3rd columns of the phylum Firmicutes in Fig 1), and *Lautropia mirabilis*, *Neisseria sp*. and *Haemophilus parainfluenzae* (1st, 2nd, and 13th columns of the phylum Proteobacteria in Fig 1). Species detected at relatively small numbers (< 500 cpm counts) include 5 species of the family Coriobacteriaceae (right-sided bold bar in the phylum Actinobacteria in Fig 1), 11 species of the family Peptostreptococcaceae [11] (third bold line from the last of the phylum Firmicutes in Fig 1), 7 species of the phylum Spirochaetes, and 4 species of the phylum TM7 (right-sided 4 columns in Fig 1). The periodontitis-associated “red complex” species *Tannerella forsythia* and *Treponema denticola* [24] were found at relatively low cpm (120.1 and 187.3, respectively), while *Porphyromonas gingivalis* was not detected in any sample. The cariogenic pathogen *Streptococcus mutans* [25] was substantially lower in subjects 1 and 2 (263.3 and 0 cpm), respectively, relative to subject 3, which exhibited moderate levels of cpm (3224.5).

A comparison of bacterial diversity among subjects showed that the species-level α diversity indicated by the Shannon's index was not remarkably different among tooth types or individuals. Among tooth types, the Shannon's index was slightly lower for incisors (average 3.81 ± 0.08 S.E.); however, values for both premolar and molar teeth were comparable (4.08 ± 0.06 and 4.17 ± 0.06, respectively). There was also little difference in the Shannon's index values among individuals (4.06 ± 0.06, 4.11 ± 0.05, and 3.88 ± 0.09 ± S.E. in persons 1, 2, and 3, respectively; S2 Fig).

### Intra-day stability and fluctuation of oral bacterial composition

**Fig 2:**
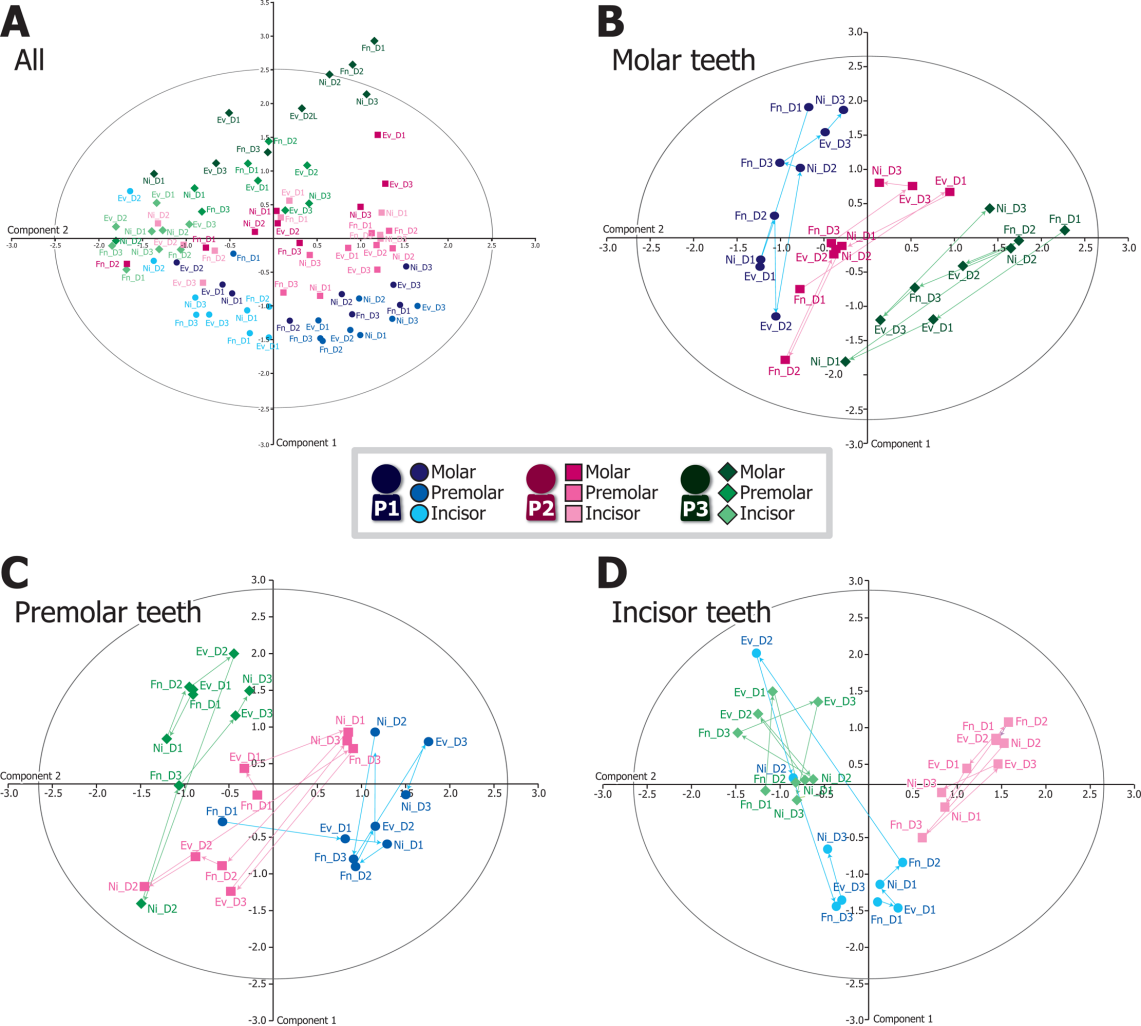
Principal coordinate analysis (PCA) of bacterial compositions of 81 plaque samples. PCA was performed using a covariance matrix of relative sequence read counts [ln(cpm)] for all 162 species across (A) all, (B) molar, (C) premolar, and (D) incisor samples. Results are shown as eigenvalue plots with 95% confidence ellipses indicated as gray-line circles. P1, P2, and P3 indicate subjects 1–3, respectively; Fn, forenoon; Ev, evening; Ni, night; D1, D2, and D3 indicate day1–3, respectively. The arrows represent the temporal sequences of the samples. The percentages of variance (%) accounted for the components 1 and 2 were 21.3 and 15.0, 25.7 and 18.3, 26.9 and 13.8, and 33.7 and 10.9 in panels A (all), B (molar), C (incisor), and D (incisor), respectively.

Next, we used principal coordinate analysis (PCA) to examine the stability of the oral bacteriome across intra-day time points, both within an individual and between subjects (Fig 2). Eigenvalue scale plots of all 81 plaque samples showed that most data points were distributed within a 95% confidence ellipse and clustered by individual (Fig 2A). Similarly, when plots were separated by tooth type (molar, premolar, and incisor; 27 data points in each category), PCA showed clear clustering by individual, with no clustering associated with sampling dates or time of day (Fig 2B–2D). In particular, molar tooth data (Fig 2B) showed non-overlapping grouping of individuals, though both intra- and inter-day changes in bacterial composition were observed in all individuals. Similar results were also obtained for both premolars and incisors (Fig 2C and 2D), though some samples did cluster with those of other individuals (*i*.*e*., forenoon sample of day 1 of person 1 and night sample of day 2 of person 3 in premolars, see Fig 2C; evening and night samples of day 2 of person 1 in incisors, see Fig 2D).

Further analysis was performed to evaluate whether bacterial species exhibited periodic fluctuations over the course of a day (S2 Table). In this analysis, significant increase or decrease of sequence counts were examined for each of the 162 bacteria by Pearson's correlation coefficients. The correlation coefficients were estimated for three time points (forenoon, evening, and night) using the data of three days, individuals, and tooth types examined. The vast majority of species exhibited no periodic changes over a course of a day, though three species (3/162; 1.85%) were found to exhibit significant changes in abundance over time. The significantly fluctuating species included *Haemophilus parainfluenzae* (*r* = −0.265, *n* = 81, *d*.*f*. = 79, *p* = 0.017; FDR (false discovery rate) = 0.003; Benjamini-Hochberg (BH) FDR < 0.05), *Cardiobacterium hominis* (*r* = 0.263, *n* = 81, *d*.*f*. = 79, *p* = 0.018; FDR = 0.003; BH FDR < 0.05), and *Clostridiales[F-2][G-2] sp*. (*r* = 0.237, *n* = 81, *d*.*f*. = 79, *p* = 0.033; FDR = 0.009; BH FDR < 0.05) (see right-sided column of the S2 Table).

### Co-occurrence and co-fluctuation relationships among bacterial species

A more detailed analysis was performed to explore co-occurrence or co-fluctuations among oral bacteria to identify species combinations and communities that are of biological, ecological, and clinical importance. As a basis of this analysis, our 81 oral samples were considered to be independent in respective to individuals, teeth, and time points, resulting in 81 unique data points (including zero cpm) for each of the 162 bacterial species. Accordingly, we can calculate correlation coefficients for each of these 81 data points across all pairs of detected bacteria. When bacteria *A* and *B* were detected higher in sample *X* (*e*.*g*., day 1 forenoon of person 1) and lower in sample *Y* (*e*.*g*., day 3 night of person 3), the correlation coefficient becomes positive, indicating a situation of co-occurrence. When the sequence counts of bacteria *A* and *B* were inversely proportional across samples, the correlation coefficient becomes negative, indicating a potential situation of antagonism or differential adaptation to environmental conditions.

**Fig 3:**
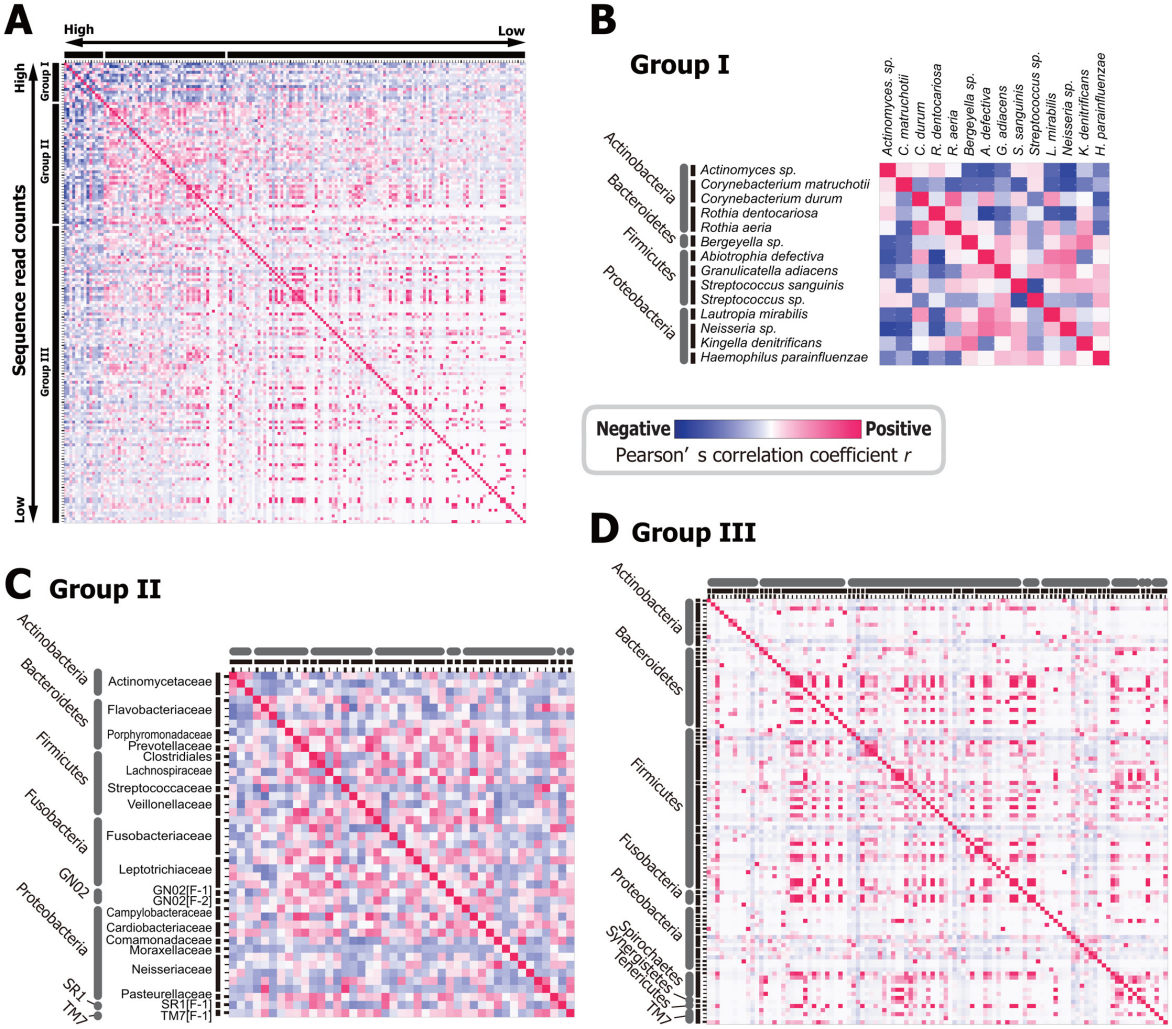
Co-occurrence or antagonism of all possible combinations of detected bacteria. Matrices colored in red shading indicate positive correlations, implying a co-occurrence between species; blue shading indicates negative correlations, implying a potential antagonism between species. (A) All 162 bacterial species detected ordered by sequence read counts. (B) Relatively abundant species (Group I), (C) moderately abundant species (Group II), and (C) less abundant species (Groups III) ordered by taxonomic hierarchies. Legend of vertical axis of the panels A, C, and D are provided as S6 Fig.

Applying this correlation analysis to the three periodically fluctuating bacteria indicated a modest degree of relation, with significant co-occurrence observed between *C*. *hominis* and *Clostridiales[F-2][G-2]* sp. (*r* = 0.259, *n* = 81, *d*.*f*. = 79, *p* = 0.019), but not between either *C*. *hominis* and *H*. *parainfluenzae* (*r* = −0.114, *n* = 81, *d*.*f*. = 79, *p* = 0.311) or between *H*. *parainfluenzae* and *Clostridiales[F-2][G-2]* sp. (*r* = −0.088, *n* = 81, *d*.*f*. = 79, *p* = 0.435). Outside of these three species, the correlations among all 162 bacteria indicated the presence of three modes of relationship (Fig 3). Relatively highly-abundant bacteria (ranging from 14,885 to 235,495 cpm across 81 samples), designated as “Group I” on the vertical axis of Fig 3A, have generally negative correlations with other species (average of −0.038; ranging from −0.488 to 0.732). This group included normal dominant bacteria commonly found in the mouth of healthy individuals, such as *Streptococcus sanguinis*, *Rothia dentocariosa*, etc. (see Fig 3B). Moderately-abundant bacteria (1,050 to 12,976 cpm) indicated as “Group II”, exhibit a slightly positive correlation with other species on average (0.079; ranging from −0.290 to 0.844). This group comprised a wide range of bacterial taxa including caries-associated *S*. *mutans* as well as other Actinobacteria, Firmicutes, Proteobacteria, and TM7 (see Fig 3C). Less-abundant or "rare" bacteria (1.5 to 1,046 cpm), including the periodontitis-associated red complex species *T*. *forsythia* and *T*. *denticola*, were designated as “Group III”. These bacteria also exhibited slightly positive correlations on average with other species (0.065; ranging from −0.148 to 1.000). However, it should be emphasized that some combinations of rare bacteria exhibited strong positive correlations (see Fig 3D; 0.950 to 1.000; colored in red), while others showed very weak to no correlation (colored in white).

**Fig 4:**
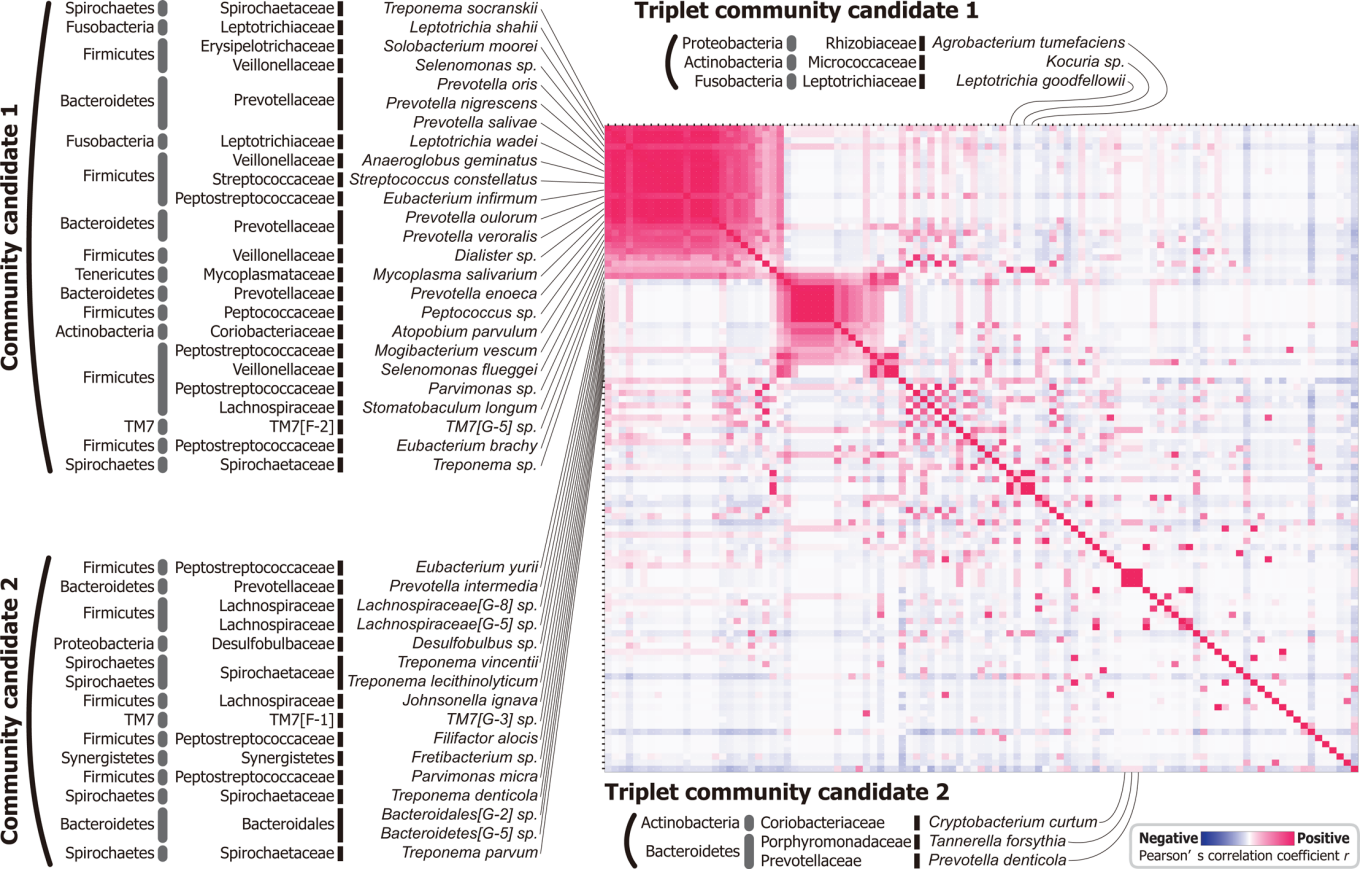
Estimation of possible bacterial communities among less abundant species. Matrices colored in red shading indicate positive correlations, implying a co-occurrence between species; blue shading indicates negative correlations, implying a potential antagonism between species. Based on the same data of Fig 3D, bacterial species were ordered by summed scores of correlation coefficients across relevant combinations, with a few manual modifications for visualization purpose.

By focusing on the group III rare bacteria (Fig 3D), we estimated the potential interactions or communities among them (Fig 4). A heatmap analysis uncovered possible bacterial communities comprised of species' combinations with relatively strong correlation coefficients between one another. These communities appeared as two relatively large and two small clusters in Fig 4, designated as "community candidate 1 and 2" and "triplet community candidate 1 and 2", respectively. The community candidate 2 and the triplet community candidate 2 included periodontitis pathogens *T*. *denticola* and *T*. *forsythia*, respectively.

## Discussion

In this study, we found that species-level bacterial composition exhibits clear differences between individuals in supragingival plaques (Fig 2) collected at three time points (forenoon, evening, and night) across three non-consecutive days from three adults. Major bacterial species tend to be common between individuals and exhibit similar composition patterns (Fig 1), while the total bacterial compositions including minor species clustered by individual in PCA plot (Fig 2). This pattern of clustering was reproducible across tooth types (Fig 2B–2D), highlighting the differential bacterial compositions present between individuals. In other words, they act as a sort of microbiological fingerprint for each particular subject, though our finding and conclusion are limited due to a smaller number of subjects. Several studies have observed differences in oral bacterial composition among teeth and gingival locations within an individual [26], [27]. Thus, a precise sampling of oral plaques is necessary to obtaining data that accurately represent an individual’s microbiome [27]. Here, we performed precise sampling of oral plaques by a dentist, and observed reproducibly distinct oral bacterial compositions among individuals over consecutive time points (Fig 2).

We also observed intra-day variability in bacterial compositions within individuals (Fig 2); however, these fluctuations did not appear to affect the patterns in which individuals cluster (see Fig 2B–2D). The remarkable exception to this observation was the day 2 incisor sample of person 1 (Fig 2D), which deviated markedly from the other samples collected from this individual. One possible explanation for this aberrant clustering might be the smaller number of usable sequences obtained for this sample (2,062 sequences; S1 Table), resulting in a biased species composition and sample misclustering. To address this possibility, we randomly chose 2,062 sequences from each sample (downsampling), and re-analyzed them in the same manner. The obtained PCR plot showed essentially same results (S3 Fig); the samples generally clustered by persons, and the day 2 incisor sample of person 1 deviated from the personal cluster. Accordingly, this additional result implies possible cross-contamination of relevant incisor samples, or relative instability of bacterial composition of incisor teeth as representative characteristics of persons. On the other hand, most of the samples, particularly molar and premolar samples with higher DNA concentrations, provided higher number of usable 16S rRNA sequences (S4 Fig), and clustered by individual in general, although intra-individual variations also exist across sampling times and tooth types. Our results are the first one to extend the view that the human oral microbiome is largely stable in one individual and differs among individuals [11], [14] to the level of intra-day time scale (Figs 1 and 2).

Further analyses revealed intra-day fluctuations for several bacterial species (S2 Table). While the majority of bacteria species appeared to not be periodically fluctuating over time, three species (1.85%; 3/162) exhibited significant and reproducible intra-day fluctuations: *H*. *parainfluenzae*, *C*. *hominis*, and *Clostridiales[F-2][G-2] sp*. (see right-sided column of the S2 Table). In particular, *H*. *parainfluenzae*, a species known to play a role in nitrogen metabolism [28] decreases significantly during the evening and night. Such intra-day fluctuations may correspond with periodic changes in the oral environments, such as availability change of nitrogen compounds. However, the estimated correlation scores were modest (−0.265 to 0.263), as they were for the other two statistically significant species. From this, we concluded that periodic fluctuations in sequence abundance are uncommon for most human oral bacteria over short periods of time. This observation is different from that of gut microbiomes and host biomolecules [15–17], though this discrepancy may be due to the smaller number of sampling time points used in this study (three times per day). By increasing the number of sampling points to include intra- and inter-day, week, and month scales, periodic fluctuations may become evident for a greater number of species than described here.

A more detailed examination of co-occurrence and co-fluctuations highlights possible functional and ecological interactions of oral bacteria (Figs 3 and 4). Among the periodically fluctuating bacteria (*H*. *parainfluenzae*, *C*. *hominis*, and *Clostridiales[F-2][G-2] sp*.), the degree of co-occurrence or antagonism relationships was modest, and did not reach statistical significance. Accordingly, these species might act independently in terms of the underlying mechanisms guiding their daily fluctuation. On the other hand, it is notable that bacterial combinations could be separated into three groups (Group I–III in Fig 3) based upon sequence abundance and their mode of interaction (co-occurrence or antagonism). The first important finding is in Group I. These highly abundant species tend to have a negative impact on the abundance of other species (blue shading in Fig 3). Such an interaction could be interpreted as a results of competition for finite space and resources, as Group I includes many of the most abundant and dominant species of the human mouth such as *Streptococcus sanguinis*, *Rothia dentocariosa*, and others (see Fig 3B) [29]. Another important observation is found in less-abundant rare species (Group III in Fig 3), many of which exhibit strong co-occurrence with other Group III species (red shading in Fig 3D). Such remarkable correlations suggest the existence of functional and/or physiological interactions among these rare bacteria.

Our analysis of the rare Group III bacteria suggests the potential interacting communities, which are supported in part by previous reports (Fig 4 and Table 1). These candidate communities are identified as a set of bacterial combinations that exhibit high and positive correlation coefficients indicative of co-occurrence (Fig 4), and suggestive of possible functional interactions. In fact, “community candidate 1” includes Fusobacteria, Veillonellaceae, and Streptococcaceae species that were proposed to be partners of coaggregation with each other [30–31]. Accordingly, a part of the community candidate 1 might reflect a plaque formation and the associated bacterial species. Furthermore, forty of the bacterial combinations shown in Fig 4 have been identified in cases of the dental caries, periodontitis, and other conditions (Table 1), mainly by checkerboard DNA-DNA hybridization and/or PCR-based analysis of dental patients [32], [33]. While many of these interactions are also evident in this study, it should be emphasized that our analysis included only healthy subjects. This suggests the potential usefulness of our metagenomic approach and analytical scheme for exploration of unidentified but possibly important bacterial combinations. For example, the bacterial communities indicated in Fig 4 may exhibit cooperative biological interactions related to activities such as metabolism or immune evasion. In addition, the constitutive species of these communities are also of interest for functional investigations because the communities include periodontitis-associated bacteria (*e*.*g*., *T*. *forsythia* and *T*. *denticola*). Future research will be necessary to validate these interactions and to determine the underlying mechanisms of action.

**Table 1:** Bacterial combinations/communities implied by pairwise correlation analysis and support by previous reports.

Bacterial combinations	Relevant pathological and/or physiological conditions	References
Community candidate 1		
*Treponema socranskii*, *Streptococcus constellatus*, *Prevotella nigrescens*, *Selenomonas*	Adult Down syndrome periodontitis	[32]
*Treponema socranskii*, *Prevotella nigrescens*	Periodontal disease and heat-shock protein production	[33]
	Endodontic infections	[46], [47]
	Root canal infection with periapical lesions	[48]
*Solobacterium moorei*, *Mycoplasma salivarium*	Refractory periodontitis	[49]
*Solobacterium moorei*, *Atopobium parvulum*	Halitosis	[50]
*Prevotella nigrescens*, *Streptococcus constellatus*	Periodontal disease, extracellular DNase secretion	[51]
	Plaque accumulation of dorsum of the tongue	[52]
	Acute periradicular abscesses	[53]
	Subgingival plaque of periodontitis patients	[24]
*Prevotella nigrescens*, *Prevotella veroralis*	Tet-Q (tetracycline-resistance gene) probe positive	[54]
Community candidate 2		
*Prevotella intermedia*, *Treponema lecithinolyticum*	Chronic periodontitis	[55]
*Prevotella intermedia*, *Treponema denticola*	Periodontal pathogens in saliva of a severe periodontitis	[56]
	Epstein-Barr virus (EBV)-associated periapical periodontitis	[57]
*Prevotella intermedia*, *Parvimonas micra*	Candidate species to promote TM7 growth	[58]
*Prevotella intermedia*, *Filifactor alocis*, *Parvimonas micra*	Peri-implantitis	[59]
*Parvimonas micra*, *Filifactor alocis*	Refractory periodontitis	[49]
	Periodontitis	[60]
*Prevotella intermedia*, *Filifactor alocis*, *Treponema denticola*	Canals of root-filled teeth with periapical lesions	[61]
*Treponema denticola*, *Filifactor alocis*	Infected root canals	[62]
	Periodontal disease	[63]
*Prevotella intermedia*, *Parvimonas micra*	Acute periodontal lesions	[64]
	Aggressive and chronic periodontitis	[65]
	Endodontic infections of primary teeth	[66]
	Candidate species to promote TM7 growth	[58]
*Prevotella intermedia*, *Treponema denticola*	Aggressive and chronic periodontitis	[67]
	Periodontal diseases in adolescents	[68]
	Primary dentition infections	[69]
*Treponema vincentii*, *Treponema denticola*	Periodontitis	[70]
*Treponema denticola*, *Filifactor alocis*, *Parvimonas micra*	Chronic periodontitis	[35]
	Necrotic primary teeth	[71]
Triplet community candidate 1		
*Agrobacterium tumefaciens*, *Kocuria sp*.	Antagonistic activity towards *Phytophthora nicotianae*	[72]
Triplet community candidate 2		
*Cryptobacterium curtum*, *Prevotella denticola*	Chronic periodontitis	[73]
*Tannerella forsythia*, *Prevotella denticola*	Periodontal disease	[63]

As with all studies using a metagenomic/metabarcoding approach, the present work is not without limitations. One important limitation is the relatively poor resolution or potential errors of species-level taxonomic assignment on the basis of shorter maker sequences of 16S rRNA (258 or 259 bp in this study) [34]. The second is that, while sequence read counts have been interpreted as quantitative, these numbers may not be truly reflective of biological abundance. Factors such as differences in the number of 16S rRNA paralogs, amplification efficiency due to GC-content, and hybridization efficiency of PCR primers for different bacterial species [35] are likely to affect sequencing outcomes. Because of these confounding factors, our data cannot be considered absolutely quantitative. Nonetheless, the species-level bacterial compositions of plaques clustered by person (Fig 2), indicating that any technical biases are likely to be reflected in all samples.

One more potential problem is in the statistical analysis of the compositional data. There has been known that standard correlation analysis (*e*.*g*., Pearson's *r* and Spearman's *r*_s_) applied to relative count data produces unreliable results, in particular between high- and low-frequency components leading to false negative correlation [36]. In fact, the average of correlation *r* was −0.030 between highly abundant species (Group I in Fig 3A) and less abundant species (Group III in Fig 3A). This value became positive (0.113) when we applied the program SparCC (Sparse Correlations for Compositional data) [36], which estimates corrected correlation coefficients based on the assumptions that when the number of components (*i*.*e*., bacterial species) is large, the true correlation network is sparse (*i*.*e*., most species are not strongly correlated with each other). Consequently, a part of negative correlation shown in the Fig 3A might be an artifact. On the other hand, most of the species (93.6%; 44/47) constituting potential rare-bacteria communities (Fig 4) were also constitute potential communities after the application of the SparCC (S7 Fig).

A caution is further required, however, in viewing and interpreting the results of correlation analysis of rare bacteria (Fig 4), which is inherently biased to detect positive correlation (co-occurrence) and less sensitive to negative correlation (antagonizing effects or differential adaptation to an environment). This is because the positive correlation can be detected when a particular combination of rare bacteria was detected in one or few samples. On the other hand, negative correlation is found when the sequence abundances of a combination of bacteria were oppositely detected in at least two samples. In addition, multiple samples of time courses from limited number of subjects may also lead to false positive correlations; *i*.*e*., bacteria combinations present in only one individual might indicate positive correlation by repeated samplings. To overcome these potential problems, a more effective or corrected method to detect true correlations among rare bacteria would be needed with increased number of subjects in future researches.

Considering potential design implications for future dental microbiome studies, we propose that oral plaque samples are largely representative of the host bacterial microbiome regardless of time of day. Case-control and disease association analyses would therefore be applicable to most oral bacterial species in general, with the notable exceptions of periodically fluctuating species *H*. *parainfluenzae*, *C*. *hominis*, and *Clostridiales[F-2][G-2] sp*. When these species are investigated, it would be preferable to conduct carefully time-controlled plaque sampling to account for such fluctuations. In terms of sample sites, our results imply that molars better reflect individual differences in the oral microbiome (Fig 2B), likely due to the higher amount of plaque DNA present in these locations (S1 Table), resulting in a higher diversity of detectable bacterial species (S2 Fig). As part of the Human Microbiome Project, oral samples were taken from six teeth (incisor, premolar, and molar from both upper and lower jaws) [6]. However, such sampling may often be beyond the means of usual laboratories in terms of facilities, funding, and manpower. In instances in which sample collections must be limited, molar plaque may be the effective target. In summary, our findings provide important insights into the design of future dental microbiome studies, particularly when a large cohort approach is taken. More expansive analyses are necessary to establish whether the observations and implications of this study are generalizable.

## Materials and Methods

### Ethics statement

All relevant research protocols and procedures were approved by Ethics Committee of Tohoku University School of Medicine, Sendai, Japan. All adult subjects provided written informed consent.

### Subjects and sampling

Three healthy Japanese males, whose ages ranged from 35 to 40 years, provided oral plaque samples. Prior to collection, subjects were confirmed to have no severe caries, periodontal disease, and any other dental disorders by Akito Tsuboi (A.T.). The DMFT (Decayed-Missing-Filled Teeth) index of the subjects was 7.7, including wisdom tooth (max = 32). The detailed clinical data at the moment of sampling was as follows; person 1, DMFT = 0 (D = 0, M = 0, F = 0), # of teeth = 29 (# of wisdom teeth = 2, left lower permanent canine and lateral incisor are fused), and active caries = 0; person 2, DMFT = 14 (D = 1, M = 0, F = 13), # of teeth = 29 (# of wisdom teeth = 1 (one wisdom tooth restored)), and active caries = 1; person 3, DMFT = 9 (D = 0, M = 0, F = 9), # of teeth = 29 (# of wisdom teeth = 1 (one wisdom tooth restored)), and active caries = 0. Supragingival plaques were collected from three lower (mandibular) teeth: a molar (#19 under the Universal Numbering System), a premolar (#28), and an incisor (#25); in one subject, molar tooth samples were collected from tooth #18, as tooth #19 was fully crowned.

Oral plaque sampling was performed for three times daily (forenoon, evening, and night) with three-day replicates (4, 7, and 10 August, 2014) at 2-day intervals. Sampling was conducted at 11:00, 16:00, and 21:00, which were intended to be at least 4 hours after the most recent meal (7:00, 12:00, and 17:00). After each meal, donors brushed their teeth. Thus all samples are 4 hours long, generally immature (early) plaques. Oral plaques were sampled by A.T. by using a sterilized curette, dissolved in 0.5 mL of Tris-EDTA (10 mM Tris and 1 mM EDTA; pH 8.0), and stored at −30°C until samples processing.

### DNA extraction

Standard glass bead-based homogenization and silica-membrane spin-column methods were applied for DNA extraction from oral plaques. A one-tenth volume of glass beads (0.1-mm diameter; AS ONE, Osaka, Japan) were added to the dissolved sample tube, and homogenized using the Mixer Mill MM 400 (Retsch, Haan, Germany) for 10 min at 30 Hz. After brief centrifugation and supernatant removal, the collected plaque homogenate (~0.25 mg including glass beads) was subjected to DNA extraction using the PowerSoil DNA Isolation Kit (MO BIO, Carlsbad, CA, USA) according to the manufacturer's protocol. DNA was eluted from the spin column in 40 μL of RNase-free water (Takara, Shiga, Japan), and stored at−30°C after confirmation of the concentration and quality using the Nanodrop (Thermo Scientific, Wilmington, DE, USA).

### PCR amplification and amplicon sequencing of bacterial 16S rRNA V4 region

Using the extracted oral plaque DNA as a template, a partial bacterial 16S rRNA fragment was amplified in a two-step PCR. First, a partial sequence of the hypervariable V4 was amplified simultaneously adding sequencing priming sites and random hexamer nucleotides (N{6}) for base call calibrations of a MiSeq platform (Illumina, San Diego, CA, USA). The length of the target region is typically 258 or 259 bp, and was amplified using primers 5′-ACACTCTTTCCCTACACGACGCTCTTCCGATCTNNNNNNGTGCCAGCMGCCGCGGTAA-3′ (forward) and 5′-GTGACTGGAGTTCAGACGTGTGCTCTTCCGATCTNNNNNNGGACTACHVGGGTWTCTAAT-3′ (reverse). First-round PCR conditions were as follows: 94°C for 3 min followed by 35 cycles at 94°C for 45 sec, 50°C for 1 min, and 72°C for 1 min 30 sec. In the second round of PCR, the first-round PCR products were amplified using the above sequencing priming sites as PCR priming sites by adding dual-index tag sequences (D5 and D7 series; Illumina) and flowcell binding sites of the Illumina adapter. The primer sequences were 5′-AATGATACGGCGACCACCGAGATCTACACD{D501 to D508}ACACTCTTTCCCTACACGACGCTCTTCCGATCT-3′ (forward) and 5′-CAAGCAGAAGACGGCATACGAGAT{D701 to D712}GTGACTGGAGTTCAGACGTGTGCTCTTCCGATCT-3′ (reverse). Second-round PCR conditions were as follows: 98°C for 30 sec, 12 cycles at 98°C for 40 sec, 65°C for 30 sec, and 72°C for 30 sec, followed by 1 cycle at 72°C for 5 min.

The tag-indexed PCR products obtained above were subjected to multiplex amplicon sequencing using the MiSeq. Tagged and barcoded PCR products were quantified using the Qubit 2.0 Fluorometer and dsDNA HS Assay Kit (Life Technologies, Carlsbad, CA, USA). Samples were then pooled in equal amounts, purified by 2% L03 agarose gel (TaKaRa) electrophoresis followed by extraction using a MinElute Gel Extraction Kit (Qiagen, Hilden, Germany), and then re-quantified using the Qubit fluorometer. Finally, 10 pM of the pooled sample was sequenced by 250 bp paired-end sequencing protocol using the MiSeq sequencing reagent kit v2 (Illumina) according to the manufacturer's instructions. The required volume molarity was estimated based on the pooled DNA concentration and assumption that the total amplicon size (including Illumina adaptors) was 402 bp and the molecular weight of a nucleotide pair (1 bp) was 660 g.

### Primary data processing and quality control of amplicon sequences

Raw sequences of bacterial 16S rRNA V4 amplicons (DRA accession number is DRA003069) were subjected to primary data processing including low-quality tail base trimming, paired-end read assembly, and primer sequence removal. First, the total data-quality of each sequencing run was evaluated based on the merged sequence data of forward and reverse reads using FastQC (http://www.bioinformatics.babraham.ac.uk/projects/fastqc/) and SUGAR [19]. After confirming that any technical errors have not occurred during the sequencing, low-quality tail sequences of each read were trimmed using DynamicTrim, available in the SolexaQA software package [37]. The cutoff threshold value was set to a Phred quality score of 10 (10^−1^ error rate) [38]. Tail-trimmed paired-end reads (forward and reverse) were then assembled using FLASH [39]. The assembled sequences were filtered using custom Perl scripts to remove reads containing N-bases or having abnormal sequence lengths (an acceptable range was set to 302 ± 10 bp). Finally, TagCleaner [40] was used to remove any remaining head- and tail-sequences derived from degenerate primers of bacterial 16S rRNA V4 (forward: 5′-GTGCCAGCMGCCGCGGTAA-3′; reverse: 5′-GGACTACHVGGGTWTCTAAT-3′). In this final step, the sequence data was transformed from FASTQ [41] to FASTA format, and subjected to sequence-based taxonomic assignments and comparative diversity analysis. Custom Unix shell and Perl scripts were used in a part of these analyses.

### Taxonomic assignments and diversity analyses of oral bacteria

The assembled amplicon sequences that had passed all of the quality filtering steps described above (typically 258 or 259 bp) were subjected to similarity-based taxonomic assignments to estimate the species composition and diversity measures of oral bacterial populations. First, redundant sequences were merged into a single sequence using the program Uclust (command derep_fulllength) [42]. In this step, singleton reads identified only once in a sample were discarded from the data set. Next, sequences exhibiting ≥ 99% identity were clustered into one representative sequence using Uclust (command cluster_otus). These sequences clustered at approximately species-level resolution were then explored for possible PCR chimeras using the Uchime software [43] and ChimeraSlayer database [44]. Custom Unix shell and Ruby scripts were used in a part of these analyses.

Following chimera exclusion, representative sequences were subjected to realignment analysis with the initial sequence data excluding singleton reads using Uclust (command usearch_global) with a similarity threshold of 99%. This analysis aimed to quantify and incorporate the number of slightly diverged sequences (1 to 2 substitutions per sequence) compared to the representative sequences. Such slightly divergent sequences include intra-species variation (polymorphism) and/or possible PCR and sequencing errors. The obtained representative sequences with information of read-count annotations were subjected to local Blastn searches against bacterial 16S rRNA sequences of the Human Oral Microbiome Database (HOMD) [23] using the NCBI Blast plus program [22]. The top hit for each sequence filtered by *E*-value threshold of *e* ^-125^ was used as the species/taxonomic annotation of each representative sequence. Species names and read counts were then transformed to the relative read counts (cpm).

The PCA plot was generated by using the software PAST [45] based on covariance matrix for samples about relative read count (cpm) of each bacterium calculated by comparison with their average values across samples. Using the matrix, the eigenvalues were calculated by the singular value decomposition (SVD) algorithm to give a measure of variance accounted for corresponding components [45]. The eigenvalues of top two components were used to depict the PCA plots. The PAST was also used to estimate α-diversity of bacterial compositions of the samples indicated by the Shannon’s index. Daily fluctuation of each bacterium composition was analyzed by Pearson's correlation coefficient calculated from time durations from initial sampling (forenoon) to second (evening; 5-hours later) and third (night; 10-hours later) sampling as explanatory variables, and the observed relative read counts (cpm) as dependent variables, across all teeth and persons. In other words, it examined whether a significant increase or decrease of each species' abundance replicated in three days and three persons. To denote FDR scores, null distribution of the correlation coefficients was estimated from 10,000 replications of random selection of the bacteria species and shuffling of its read count matrix based on the Mersenne Twister algorithm using a custom Perl script.

## Supporting Information

S1 Fig Estimated bacterial compositions across four sequencing replicates. (PDF)

S2 Fig Species-level α diversity as measured by Shannon's index for all 81 samples included in this study. (PDF)

S3 Fig Principal coordinate analysis (PCA) of bacterial compositions of 81 plaque samples based on randomly chosen 2,062 sequences from each sample. (PDF)

S4 Fig Correlation between sample DNA concentration and number of usable sequence reads. (PDF)

S5 Fig Legend of horizontal axis of the Fig 1. (XLSX)

S6 Fig Legend of vertical axis of the panels A, C, and D of the Fig 3. (XLSX)

S7 Fig Possible bacterial communities among less abundant species estimated based on corrected correlation analysis by using SparCC program. (XLSX)

S1 Table Number of reads remaining after each pre-processing step. (XLSX)

S2 Table Evaluation of possible periodic increases or decreases in each bacterial species across three sampling days. (XLSX)
