# Supplementary figures and images for: Inter-Individual Differences in the Oral Bacteriome Are Greater than Intra-Day Fluctuations in Individuals
Source: PLoS One. 2015 Jun 29;10(6):e0131607. doi: 10.1371/journal.pone.0131607 (PMC4487993; doi:10.1371/journal.pone.0131607)

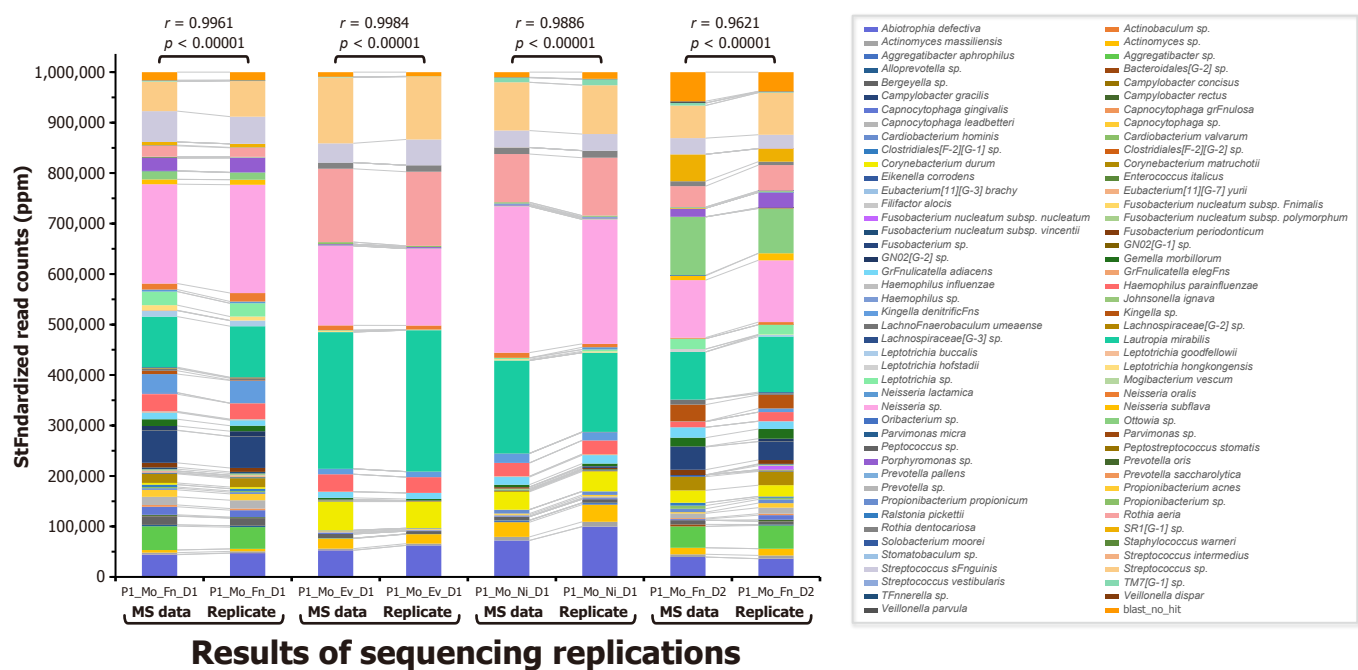

Sato *et al.* Supporting Information Figure S1

Supplement: S1 Fig — (PDF) [file pone.0131607.s001.pdf]

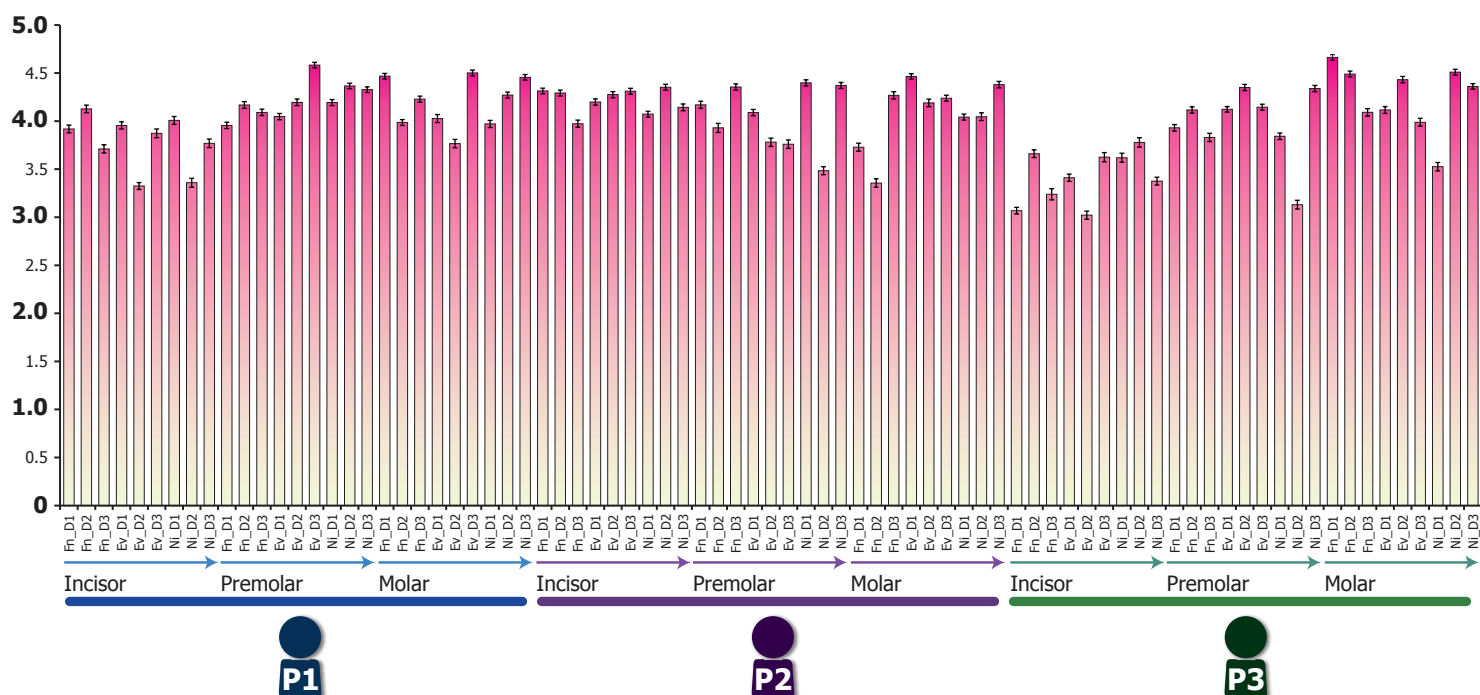

**Alfa diversity**  
(Shannon index; 10,000 bootstrap)

Supplement: S2 Fig — (PDF) [file pone.0131607.s002.pdf]

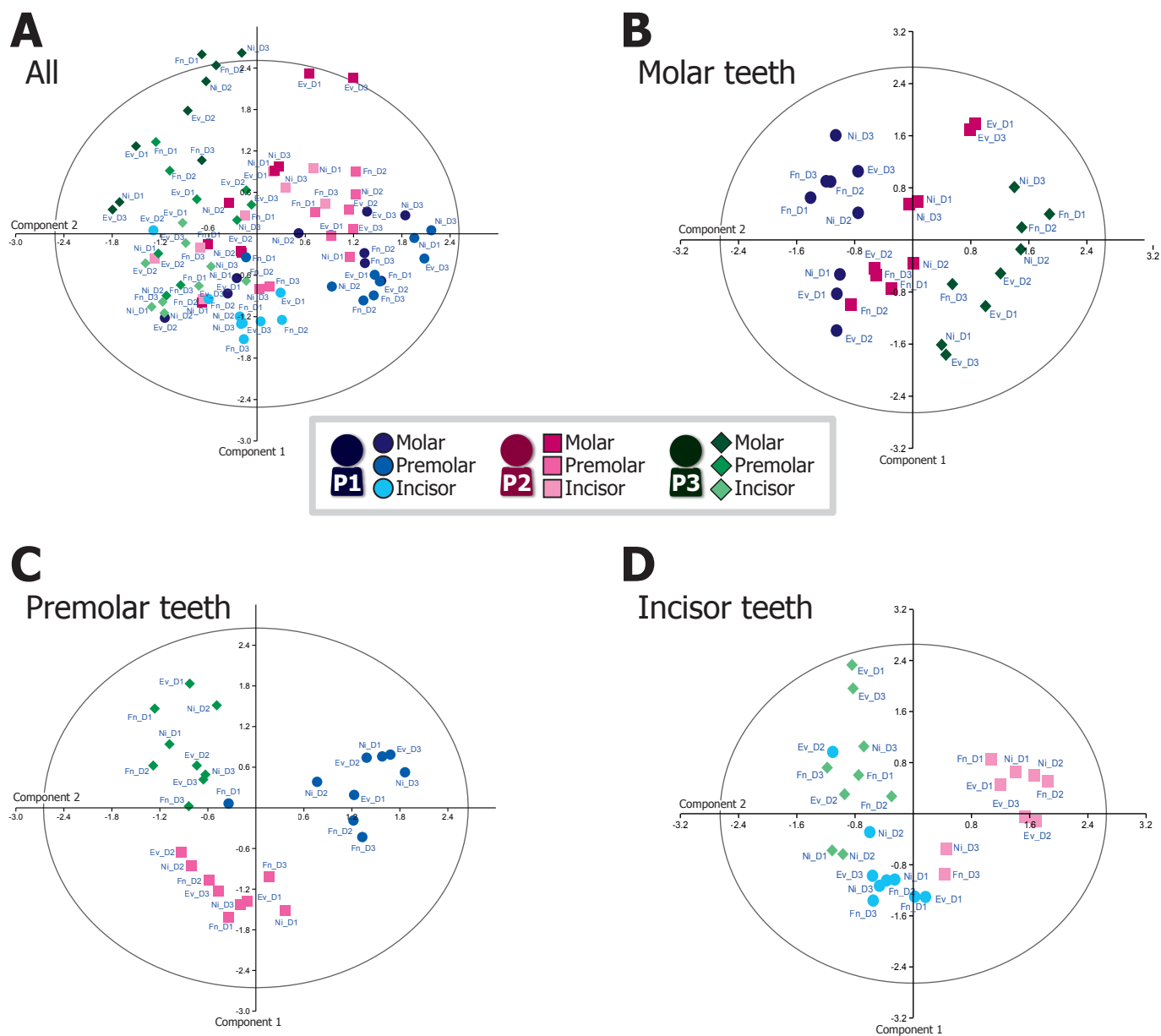

Sato *et al.* Supporting Information Figure S3

Supplement: S3 Fig — (PDF) [file pone.0131607.s003.pdf]

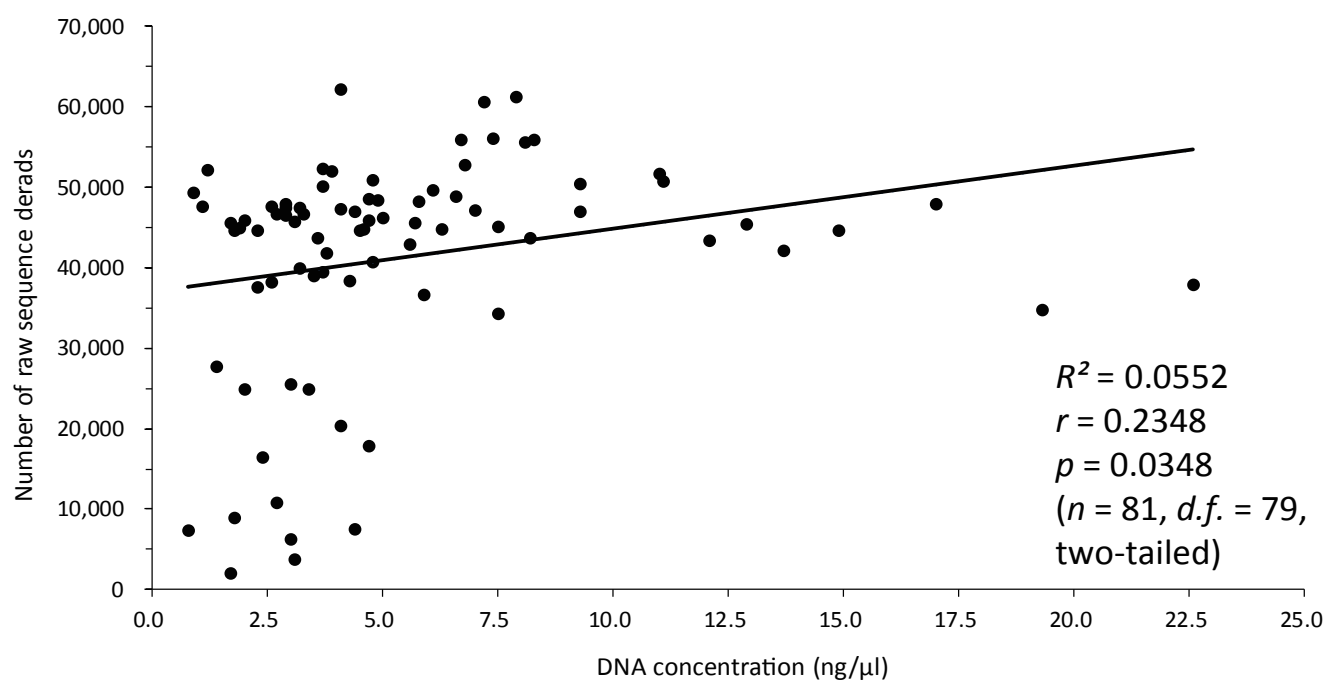

**Correlation between sample DNA concentration  
and number of usable sequence reads**

Supplement: S4 Fig — (PDF) [file pone.0131607.s004.pdf]
